# Stationary beam full-field transmission helium ion microscopy using sub-50 keV He^+^: Projected images and intensity patterns

**DOI:** 10.3762/bjnano.10.160

**Published:** 2019-08-07

**Authors:** Michael Mousley, Santhana Eswara, Olivier De Castro, Olivier Bouton, Nico Klingner, Christoph T Koch, Gregor Hlawacek, Tom Wirtz

**Affiliations:** 1 Advanced Instrumentation for Nano-Analytics (AINA), MRT Department, Luxembourg Institute of Science and Technology, 41 rue du Brill, L-4422 Belvaux, Luxembourg; 2 Institute of Ion Beam Physics and Materials Research, Helmholtz-Zentrum Dresden-Rossendorf e.V., Bautzner Landstr. 400, 01328 Dresden, Germany; 3 Department of Physics, Humboldt University of Berlin, Newtonstraße 15, 12489 Berlin, Germany

**Keywords:** charging, helium ion microscopy, ion diffraction, ion scattering, transmission ion microscopy

## Abstract

A dedicated transmission helium ion microscope (THIM) for sub-50 keV helium has been constructed to investigate ion scattering processes and contrast mechanisms, aiding the development of new imaging and analysis modalities. Unlike a commercial helium ion microscope (HIM), the in-house built instrument allows full flexibility in experimental configuration. Here, we report projection imaging and intensity patterns obtained from powder and bulk crystalline samples using stationary broad-beam as well as convergent-beam illumination conditions in THIM. The He^+^ ions formed unexpected spot patterns in the far field for MgO, BN and NaCl powder samples, but not for Au-coated MgO. The origin of the spot patterns in these samples was investigated. Surface diffraction of ions was excluded as a possible cause because the recorded scattering angles do not correspond to the predicted Bragg angles. Complementary secondary electron (SE) imaging in the HIM revealed that these samples charge significantly under He^+^ ion irradiation. The spot patterns obtained in the THIM experiments are explained as artefacts related to sample charging. The results presented here indicate that factors other than channeling, blocking and surface diffraction of ions have an impact on the final intensity distribution in the far field. Hence, the different processes contributing to the final intensities will need to be understood in order to decouple and study the relevant ion-beam scattering and deflection phenomena.

## Introduction

The use of helium ions for microscopy and nanoanalysis is gaining popularity due to the availability of the high-brightness gas field ion source (GFIS) [1]. Due to the high brightness, the probe from a GFIS can be focused into a spot of less than 0.5 nm [1]. The short wavelength of helium ions results in a reduced diffraction limit for helium ion probes compared to electrons. Helium ions also have a smaller penetration depth than electrons of similar energy and a reduced interaction volume [2], increasing the lateral resolution. In addition to the lower energy of the secondary electron (SE) emission [3], the absence of back-scattered electrons allows imaging with a He^+^ probe to be more surface sensitive than imaging with an electron probe. For these reasons, helium ion microscopy (HIM) is increasingly being used to study a wide range of materials including inorganic surfaces [3–4], thin organic layers [6–7] and biological samples [8–10]. Similarly, imaging with neutral helium atoms has also been recently developed [11–12].

While helium is clearly a useful experimental probe, most of the past work using sub-50 keV He^+^ ions only measured signals that are emitted back from the sample surface, e.g., secondary electrons [3,13], secondary ions [14–16], back-scattered ions [5] or photons [17] with a possible combination of these signals to perform correlative microscopy [18]. The potential to use transmitted sub-50 keV helium for imaging has yet to be fully explored. Transmitted helium imaging with MeV energy has been demonstrated in the past [9–10]. However, such large-scale instruments are not widely available. In comparison, sub-50 keV He^+^ ion beams are more commonly available, especially with the commercial production of HIM instruments.

The use of sub-50 keV transmitted He^+^ ions for microscopy has already been reported [19–21]. As an example application, the secondary electrons produced by transmitted ions have been used to image hollow glass nanocapillaries [22]. Features similar to diffraction-related thickness fringes seen in transmission electron microscopy (TEM) have been observed in a HIM and have possible explanations involving diffraction [19] or inelastic scattering [23] of He^+^ ions. This observation led to computational work investigating coherent scattering, channeling and diffraction of He^+^ ions in crystals to explore the possibilities to obtain atomic-resolution images in a HIM [23]. Hence, experimental investigation of the intensity distribution of transmitted He^+^ ions can provide valuable insights related to ion–solid interaction such as channeling, blocking and diffraction [23–25] and can open new imaging modalities using transmitted He^+^ ions.

To understand the different processes contributing to the deflected intensity of transmitted He^+^ ions, we investigated BN, MgO, NaCl and Si samples using an in-house built transmission helium ion microscope (THIM). In this instrument the helium ions (or post sample atoms, after neutralization) are collected behind the sample and will have been either transmitted or deflected through a small angle. We designed and developed this dedicated microscope to allow for more experimental flexibility than is possible in a commercial HIM. A notable prior work on transmission ion microscopy was performed by Melmed and Smit [26]. Unlike the instrument presented here, their setup was based on a lens-less point projection configuration.

This paper presents THIM shadow images as well as the deflected He^+^ ion intensity patterns recorded with sub-50 keV He^+^ ions. The observed differences in the He^+^ ion intensity distribution as well as unexpected spot patterns are discussed. The results suggest that information about the sample morphology can be extracted.

The results also show that charging-related effects can significantly contribute to the final intensity distribution of deflected He^+^ ions and these effects must therefore be removed from the recorded intensity patterns in order to quantify channeling and diffraction effects.

## Experimental

The samples used in this study were powders of BN (Sigma-Aldrich Product #790532), MgO (Sigma-Aldrich Product #549649) and NaCl (Plano Product #46-2). A small amount (≈1 mg) of the powder was deposited either from a spatula (BN and MgO) or scratched with a scalpel blade (NaCl) onto TEM grids without any support membrane (finder grids from Ted Pella Product #79750, Maxtaform™ Style H2, 200 mesh Cu grids) moistened with deionized water. The moist TEM grids allowed some of the crystals to stick to the grid bars. The particles that remained attached to the grids were then imaged in the THIM instrument. After the first round of imaging, for comparison, the same MgO powder sample was sputter-coated with approximately 10 nm Au on both sides to prevent charging.

**Figure 1:**
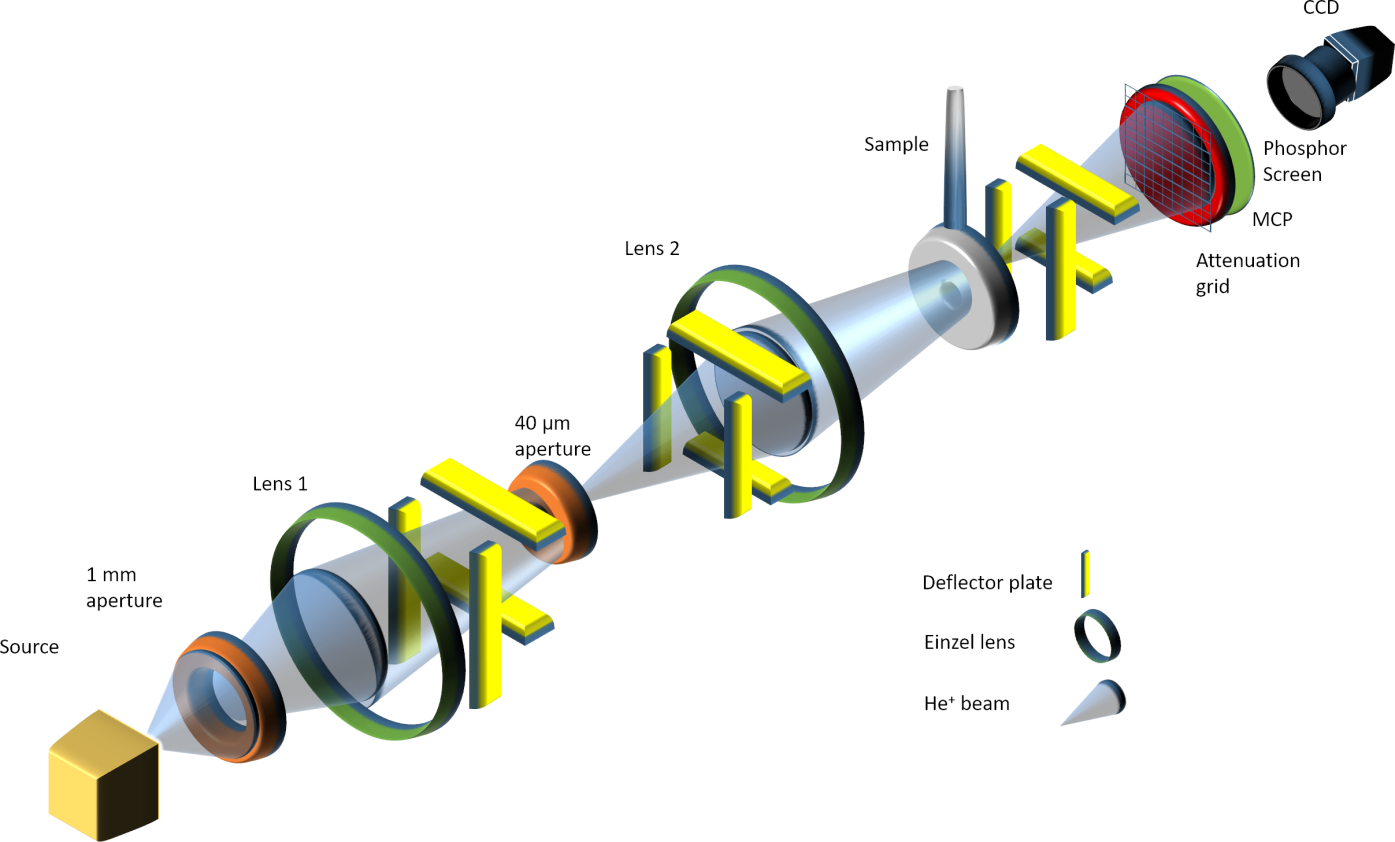
Schematic of the transmission helium ion microscope (THIM).

The THIM instrument used in this study was built in-house and is shown schematically in Figure 1. It uses a duoplasmatron source (from a Cameca IMS 4f SIMS instrument) to produce a He^+^ beam. The brightness of such a duoplasmatron source at this beam energy is in the range of 10–10^2^ A/cm^2^sr and hence seven orders of magnitude lower than that of the GFIS used on commercially available HIM instruments. This results in micrometer-sized He^+^ probes with the duoplasmatron source compared to nanometer probes with the GFIS, and the beam currents with the duoplasmatron can be three orders of magnitude higher (nA versus pA) [27]. However, the duoplasmatron current densities are around four orders of magnitude lower, 63.7 nA/mm^2^ for 2 nA in a 100 µm spot in a duoplasmatron versus 2.5 × 10^6^ nA/mm^2^ for 0.5 pA in a 0.5 nm spot in a HIM. The maximum primary ion energy achievable in the THIM prototype is limited by its power supply electronics to a value of 12.5 keV. Thus, 10 keV was chosen such that the acceleration voltage could be increased or decreased from the main operating voltage. The energy of the He^+^ was 10 keV unless otherwise stated. The ions leave the source and travel through a 1 mm differential pumping aperture. A first einzel lens then focuses the ions onto a 40 µm aperture. After the 40 µm aperture, a second einzel lens focuses the ions onto the sample (see Figure 1), from which the ions travel 78.5 cm to the detector. The detection system is comprised of a microchannel plate (MCP) in front of a phosphor screen, whose intensity is recorded with a CCD camera (Edmund Optics EO-0312M). There is a metal mesh attenuation grid in front of the MCP, which mechanically blocks 90% of the incoming intensity to prevent damage to the MCP. There are three sets of XY parallel plate deflectors in the THIM. The first two sets are after the first lens, and these are used to correct the shift and tilt when positioning the beam onto the center of the second lens. A final set of deflectors is after the sample, which were used to control the position of the post sample ion intensity on the MCP. The scale bars on the images have been estimated by calculating the size of a single pixel within the image. For this, the number of pixels across the known MCP diameter (19 mm) were counted. The experimental arrangement has a collection semi-angle of 12.1 mrad (measured from the sample to the MCP).

For comparison, complementary secondary electron (SE) imaging was performed for selected samples in a Zeiss HIM Orion Nanofab instrument. The primary energy and primary beam current were 10 keV and 0.6–0.8 pA, respectively, for HIM SE imaging.

## Results and Discussion

**Figure 2:**
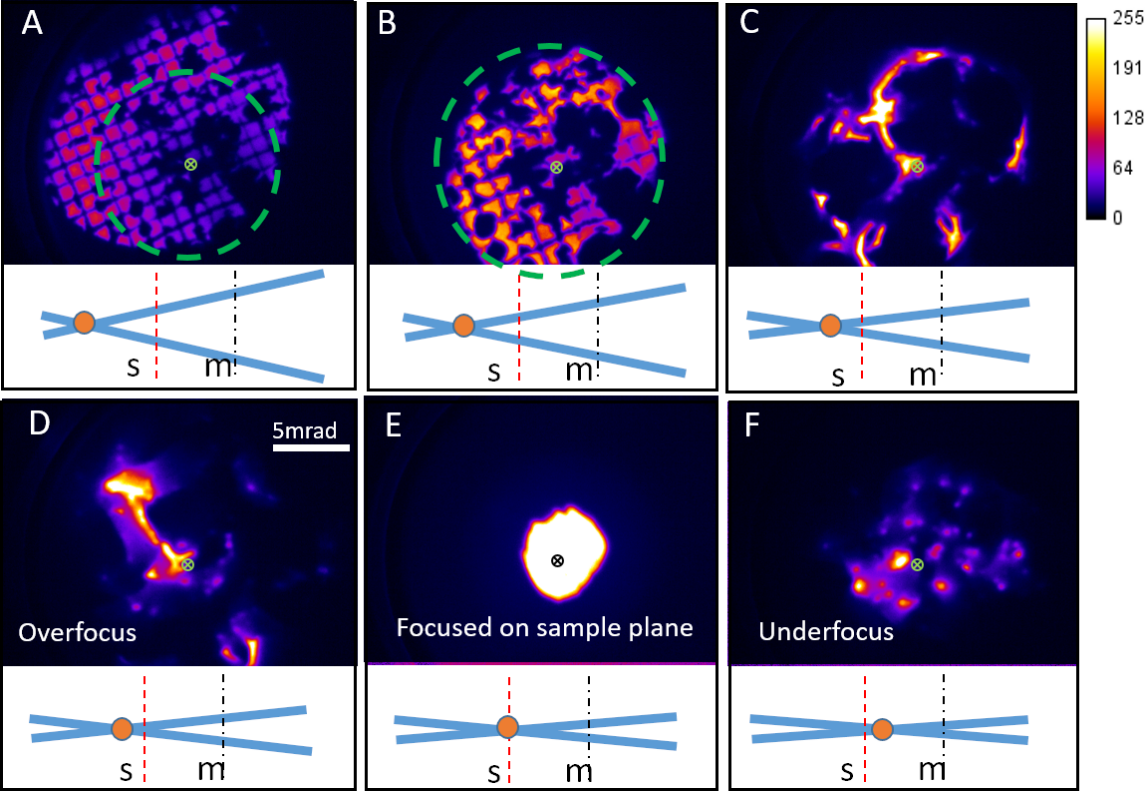
For a BN sample, the voltage of Lens 2 was decreased from (A) to (E). A) A shadow image, B) a higher magnification shadow image of the area highlighted with the green dashed circle in A, C) an even higher magnification and the start of deflection pattern formation with bright outlines, D) the overfocus deflection pattern , E) beam focused on sample plane and F) the underfocus spot pattern. The scale bar in D is 5 mrad (0.39 cm for a sample to MCP separation of 78.5 cm) and applies to all images. (The green or black crosses mark the approximate beam focal point, i.e., the beam axis). The grid window is 108 µm wide with a grid bar width of 19 µm. Underneath each image is a lens diagram showing the position of the Lens 2 focal point cross over (orange circle), the dashed red line is the sample plane (s) and the dot-dashed black line is the MCP plane (m). The color bar shows the grayscale pixel value recorded in the CCD image.

The THIM instrument shown schematically in Figure 1 was used to acquire the THIM images of BN nanoparticles adhered to a TEM grid as shown in Figure 2. A video was recorded while varying the Lens 2 voltage (which sets the ion beam focal point) from approximately 5 kV to 9 kV. Simulations with SIMION [28–29] showed that this voltage range forms focal points, i.e., images of the source spot, at approximate defocus values of +780 mm to −70 mm (see Supporting Information File 1 for details). A series of images were then extracted from the video frames. In the following section the terms overfocus and underfocus will refer to the ion beam focal point being in front of or behind the sample plane, respectively, when looking from the source to the detector. All of the THIM images are formed by direct projection of a stationary beam (diameters, in the sample plane, from approximately 2 mm to 100 µm) rather than using a scanning beam. In Figure 2A the beam is overfocused, providing broad beam illumination, which projects a shadow image of the sample onto the MCP. As the Lens 2 voltage is reduced, the ion beam focal point approaches the sample, which increases the magnification of the image obtained on the MCP while decreasing the area illuminated on the sample as shown in Figure 2B. With further reduction in the voltage of Lens 2 (as shown in Figure 2C and 2D), the magnification is increased and the intensity is redistributed from an image of the grid to a deflection pattern. The intensity is diverted away from areas of the BN powder and forms bright outlines to the darker shadowed areas, such as those surrounding the green cross in Figure 2A. These dark areas appear to distort and expand (see Figure 2 and Supporting Information File 1, Figure S4) as the beam focal point approaches the sample. Continuing to decrease the voltage of Lens 2 produces a deflection pattern (Figure 2D) when the beam is focused close to, but still in front of, the sample (slight overfocus from the sample). A further decrease in the voltage of Lens 2 then places the beam focus onto the sample plane (Figure 2E). This is an image formed by projection of the beam through the 1 mm aperture when the beam is focused onto the sample in the gap between BN particles at the point indicated by a green cross in Figure 2A. The intensity comes from a small area in the sample plane and the MCP intensity distribution reflects the angular opening of the ion beam defined by the apertures and lens system. Further reducing the Lens 2 voltage slightly underfocuses the beam, resulting in a spot pattern as seen in Figure 2F. Continuing to decrease the Lens 2 voltage produces a focused spot on the MCP (visible in the videos for BN and MgO, Supporting Information File 2,2) and then an inverted image of the sample.

The formation of these types of spot patterns was not expected. The spot patterns appear in underfocus conditions when the ion beam focal point is placed below the sample. To understand the exact reason for the formation of spot patterns seen in Figure 2, complementary investigations with other samples were carried out.

**Figure 3:**
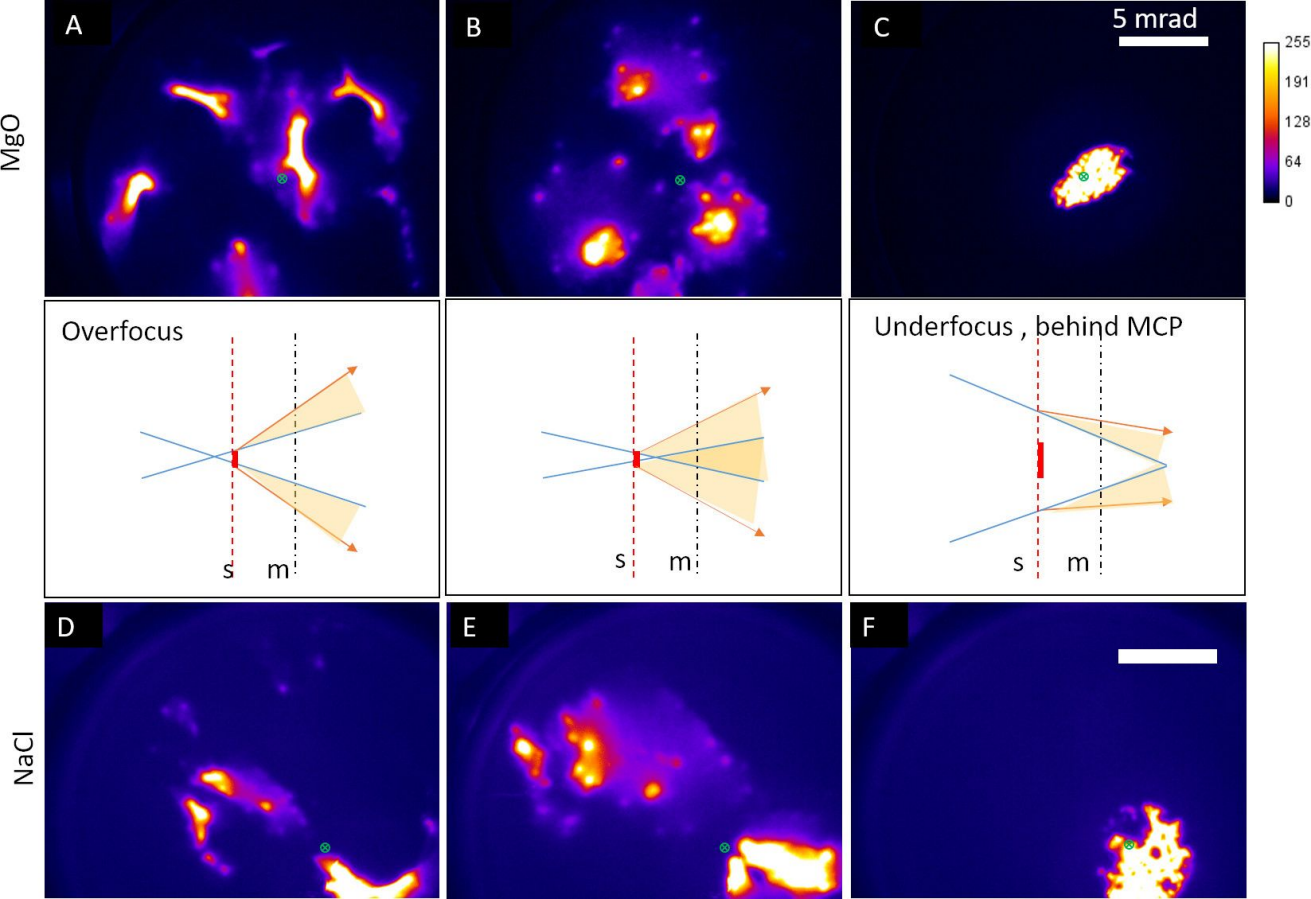
Examples of overfocus bright outline deflection patterns (A and D), underfocus spot patterns (B and E), and underfocus images (C and F) recorded for MgO (A–C) and NaCl (D–F). The scale bar in C is 5 mrad and applies to A–C. The scale bar in F is 5 mrad and applies to D–F. The color bar shows the grayscale pixel value recorded in the CCD image. The schematics in the middle row show the different deflection situations for a single positively charged particle in the path of the ion beam. The blue lines represent undeflected trajectories while the orange arrows and shaded areas show a collection of deflected trajectories. The dashed red line (s) shows the sample plane and the dot-dashed black line (m) shows the MCP plane.

The same experiment was conducted with NaCl and MgO crystal powders at similar imaging conditions, and deflection patterns were formed for both overfocus images while spot patterns were formed in all underfocus images. The results, with the Lens 2 overfocused and underfocused, are shown in Figure 3. The underfocus spot patterns can be observed for all three samples. The underfocus spot patterns consist of multiple groups of spots that enclose areas of diffuse intensity.

The appearance and behavior of the bright spots is consistent with a caustic pattern [30] where intensity is focused into the cusps of the patterns (see video Supporting Information File 3 to watch the effect of the Lens 2 voltage on MgO sample shown in Figure 3B). The limited magnification of the direct transmission mode imaging makes these cusps appear as spots on the images, while also making it impossible to see any detailed internal structure of the intensity. In addition, the angular spread of emission of the duoplasmatron source will act to blur the details in the final intensity distribution. This effect can be minimized by using a higher brightness GFIS source.

**Figure 4:**
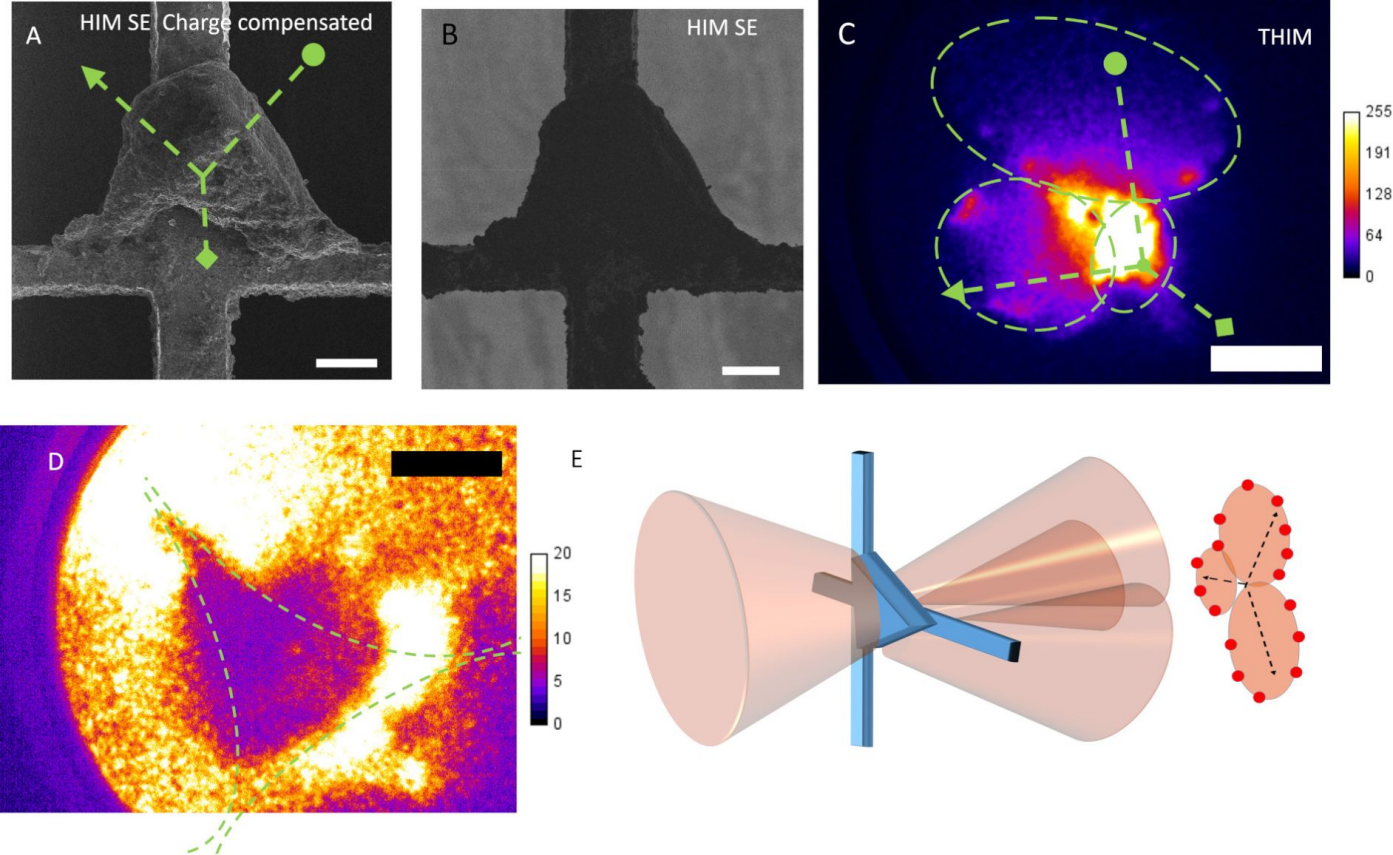
A) HIM SE image with charge compensation by electron flooding and B) HIM SE image without charge compensation. C) THIM deflection from the BN particle showing three regions of intensity outlined with dashed green ovals. D) A THIM deflection pattern with beam focus just behind the sample plane when the deflection areas are spread out. Dashed green lines outline the three separate regions of intensity. E) Schematic illustration of the deflection process. During THIM, He^+^ ions had an energy of 12.5 keV. Scale bars: (A,B) 20 µm and (C,D) 5 mrad. The set of three lines in A and C (with an arrowhead, a circle and a diamond at the ends) are rough guides for the eye showing the relative orientation of the sample and the directions of deflection and expansion (visible in Supporting Information File 2, BN video). The color bar shows the grayscale pixel value recorded in the CCD image.

To further understand the cause of the deflection pattern, a triangular BN particle was placed in a commercial HIM and SE images were recorded for comparison. The HIM SE images, taken with and without charge compensation by electron flooding are shown Figure 4A and Figure 4B, respectively. The results in this paper suggest that charging occurs both for the low current density, larger beam area of the THIM prototype as well as the high current density, small probe area of the HIM. The net steady-state magnitude of charging will depend on the balance of the specific kinetics related to charge build-up and charge dissipation processes.

To explore the possible correlation between the local surface morphology and the observed intensity patterns, the same BN sample was also imaged in the prototype THIM, where Figure 4C shows the THIM intensity pattern. Through a comparison of Figure 4A–C, it can be seen that the direction of the intensity deflection and expansion (see video for BN, Supporting Information File 2) matches the approximate local surface normal directions of the three main sides of the particle (lines are drawn as guides for the eye in Figure 4). This suggests that the orientation of the particle surface is responsible for the direction of deflection. The local surface topography then defines the caustic pattern and the exact position of the bright points around the edge. Figure 4C shows that there is still a large amount of intensity at the center of the shape, which is not due to direct transmission through the powder but instead due to the deflection from the edges. This effect is shown schematically in Figure 4E where the brightest part of the deflection pattern is where all three deflection areas overlap. This is confirmed when varying the voltage on Lens 2 to bring the focal point closer to the sample plane, going from underfocus closer to focus. This increases the intensity on the particle, increases the magnification and spreads out the deflected areas. When doing this, a triangular gap appears in the intensity distribution as shown in Figure 4D (see the BN video Supporting Information File 2 to watch this occur). The dashed green lines outline the edges of the three regions of intensity at each surface normal direction, highlighted with dashed green lines in Figure 4C.

**Figure 5:**
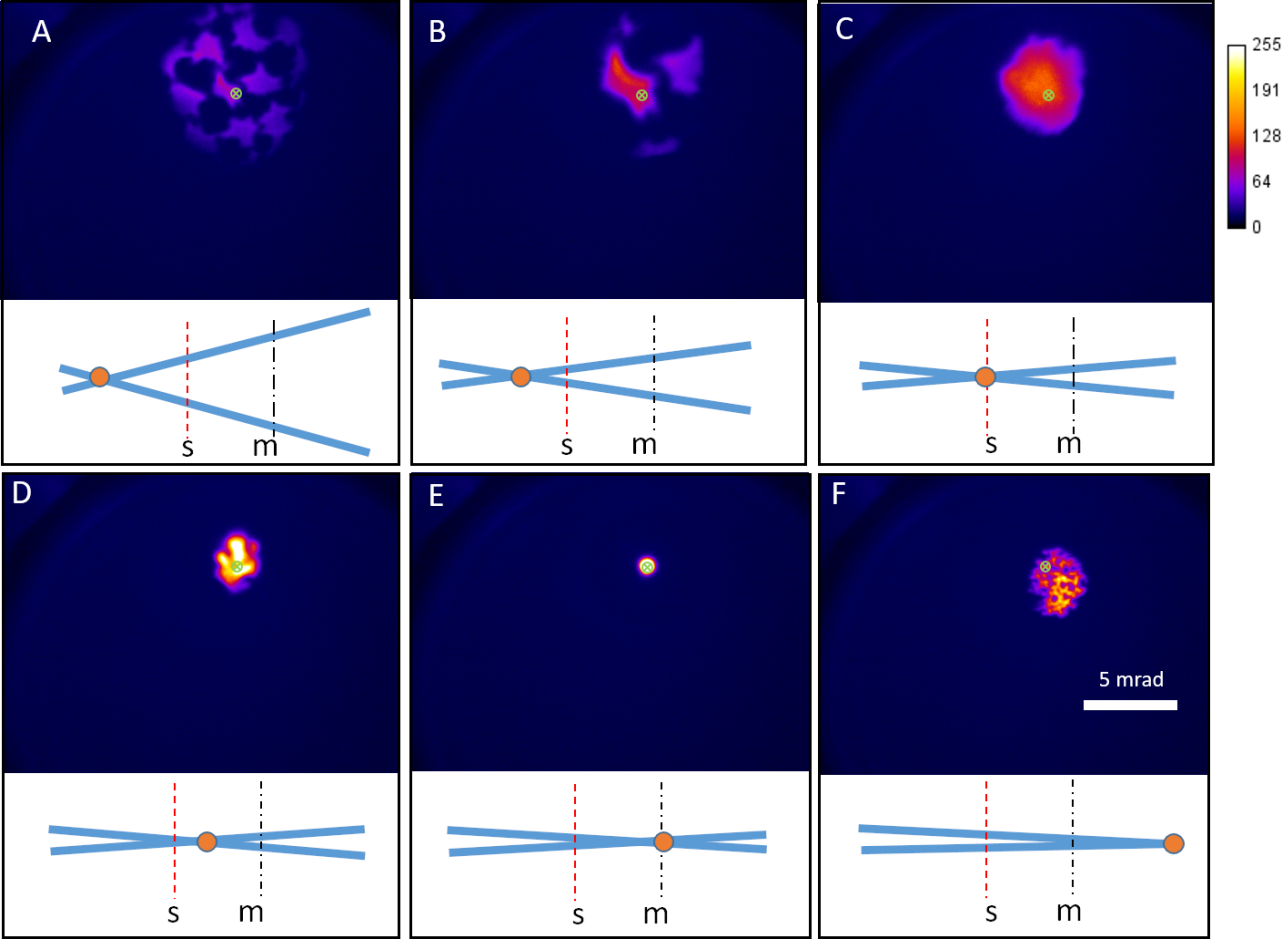
A) A THIM through-focus series of the MgO sample (coated with 10 nm Au on both sides) produced by decreasing the Lens 2 voltage from A to F. No deflection is visible, only inversion of the image occurs close to infinite magnification. Underneath each image is a lens diagram showing the position of the Lens 2 focal point cross over (orange circle), the dashed red line is the sample plane (s) and the dot-dashed black line is the MCP plane (m). The color bar shows the grayscale pixel value recorded in the CCD image. The scale bar in F is 5 mbar and applies to all images.

To confirm the role of charging in the formation of the deflection patterns, the MgO sample was coated with approximately 10 nm of Au on both sides of the sample. Figure 5A–F shows a series of images, at different defocus values, obtained by varying the voltage of Lens 2. The image recorded when the beam is focused on the sample plane is shown in Figure 5C and can be compared to Figure 2E. For both of these lens settings, the beam passes through a gap in the sample and the final image is not influenced by the structure of the sample. For both overfocus and underfocus, only a shadow image is produced. Throughout the series there are no deflection spot patterns like the ones in Figure 3 and the images are reproducible, with no distortion, when returning the lens voltage to the previously used values, showing a lack of charging. The drastic difference in results between the crystals in Figure 3 and Figure 5 shows that interaction with the charged surfaces of the powder can explain the caustic-like deformation and deflection visible in Figure 2, Figure 3 and Figure 4. The primary mechanisms for the positive sample charging are the emission of secondary electrons, secondary anions and neutralization of primary ions. In terms of charging, these mechanisms will be in competition with the resupply of electrons from the grounded Cu grid as well as the production of secondary cations. The grid material as well as the degree of contact with the grid will both affect the rate of charging. It is clear that for the powder samples this competition between charging and resupply of electrons leads to more charge remaining on the sample when the powder is not coated with metal. This increased charging then is the likely cause of the observed spot patterns.

It is interesting to note that the spot pattern of the type shown in Figure 2F only occurs when there is a cross over in between the sample and the MCP. If the focal point is moved before the sample, overfocus deflection patterns or images are seen. However, if the focal point is after the MCP, then an image is formed. The formation of expanding dark areas is a similar phenomenon to what is seen in mirror electron microscopy (MEM), where dark “bubbles” are formed in the image where the sample becomes negatively charged [31]. In MEM, when the electron beam goes through the focus, the contrast in the image can invert and dark bubbles become bright stars. The variation of the deflection pattern to a spot pattern in this experiment appears to be analogous and can be explained by considering the trajectories of ions with and without deflection. It is only when the focal point is in between the sample plane and the MCP that deflected trajectories overlap in the region behind the object. This is shown schematically in the diagrams of Figure 3. Under the condition of underfocus, but keeping the focal point in between the sample plane and the MCP, appears to be a necessary condition for producing the spot patterns. The charging, visible in Figure 4B, also explains why the proportion of the image taken up by the dark shadowed areas due to the BN powders increases from Figure 2A to 2C when the focal point is brought closer to the sample plane. The increasing beam current density on the sample increases the rate of charging and so the equilibrium charge on the sample is increased, thus deflecting the ions to a larger angle. This increased deflection for higher current densities distorts the mesh grid squares visible in Figure 2A near the green cross into the compressed intensity near the green cross in Figure 2C (see Supporting Information File 1, Figure S4 for magnified views of this distortion).

In the deflection patterns of Figure 3, there are multiple areas of bright intensity that are shifted away from the main beam axis (shown with crosses in Figure 3). The relative position of these brightest spots vary as the voltage of Lens 2 is altered (as can be seen in the video for MgO, Supporting Information File 3). This differentiates these patterns from a transmission diffraction pattern where the reciprocal lattice positions remain fixed and only the angular width of the spots changes while varying the strength of the probe-forming lens. Furthermore, the d-spacings corresponding to these spots would be unphysically small (1 pm compared to a usual range of 300 to 600 pm [32–33]). When illuminating multiple grid squares we suspect that each separate collection of spots originates from one individual grid square. The net field of the surrounding charged powder deflects the intensity to an overall direction and the exact morphology of the powder then distorts the beam into the final spot arrangement seen. The response of the spots to variations in the Lens 2 voltage (see Supporting Information File 2 and Supporting Information File 3 for videos) can be partly explained by stronger deflection from an increased rate of charging. However, the motion of the spots could also be due to the most intense section of the beam (i.e., the focal plane of Lens 2) intersecting with different positions along the surfaces of the charged sample, having varying topography, which then deflect the beam to different locations. In addition to these effects, once the charge deflects the ion beam, it prevents ions from hitting the sample, thus allowing electrons to reduce the charge until the ions hit the sample again, once more causing charging. This can be expected to cause some natural fluctuations in the spot deflection. It will be a combination of the charging rate, focal plane position and natural charge fluctuations that determines the spot positions observed at each Lens 2 voltage.

Future work on understanding these patterns could include simulations of predicted intensity from more basic systems such as individual insulating spherical particles on a metal grid. Simulations would allow easier control of the structure of the particle compared to experiments and could yield useful insights related to the role of sample morphology on the intensity redistribution.

While the spot patterns observed in this work illustrate possible artefacts, these mechanisms can offer potential applications as well. For instance, they can be useful in the characterization of micrometer-scale objects, when assessing the local surface roughness and structure. In the reports by Kiser et al. [34–35] an algorithm was used to find the structure that produces a desired intensity distribution when light is reflected or refracted from it. However, similar deflection patterns as those presented here could be used as the input (target caustic intensity) and then a reconstructed approximation to the sample surface could be obtained. Indeed, if a highly coherent He^+^ source was available, techniques similar to coherent diffractive imaging could be used. These techniques apply an iterative procedure to retrieve the phase of a wave [36]. In reflection, this phase would be determined by the surface potential, which is linked to the topology or local work function of the material, similar to studies using mirror electron microscopy [37]. There may be many solutions satisfying the intensity distribution, so, to get an approximation closer to the true surface, constraints on the solution may be required. This could be achieved by using information from other ion images such as HIM SE images or the THIM images shown here.

Finally, it should be noted that future investigations of intensity distribution from transmitted ion experiments in search of transmitted ion diffraction and other new scattering or deflection phenomena should be done carefully. An important conclusion from the present work is that spot patterns in transmitted ion intensities can contain serious artefacts, which could complicate the interpretation of images and scattered or deflected intensities.

## Conclusion

THIM-projected shadow images using a stationary 10 keV He^+^ ion beam are presented. It is shown that when the beam is focused in front of or behind powdered non-conductive samples, a 10 keV He^+^ ion beam can interact to produce detailed intensity distributions with spots of bright intensity. These patterns are similar to caustics and are not visible when a thin conductive metal coating has been deposited onto the powder. The formation of the spot patterns is explained by the deflection of ions due to electrostatic charging of the non-conductive powder. The deflection directions of the He^+^ ions have been shown to match the local surface normal direction of the sample edges. The patterns carry information on the surface morphology. However, for these powder samples there is too much information to separate many different features. Further study is required to provide a deeper understanding of the processes involved. The results presented in this paper show that there is potential to gain information about surface morphology from the spot patterns in deflected He^+^ ion images. The results presented here indicate that the final deflected intensity distribution in the far field carries contributions from sample morphology in addition to other possible phenomena such as channeling, blocking and surface diffraction of ions. Hence, the various contributions to the final deflected intensities should be understood in more detail to decouple and study the individual ion beam deflection phenomena.

## Supporting Information

File 1 The effect of varying the Lens 2 voltage on the BN deflection pattern.

File 2 The effect of varying the Lens 2 voltage on the MgO deflection pattern.

File 3 Description of supporting videos 1 and 2, SIMION results and methods (defocus and size on sample plane), a defocus series for a polycrystalline silicon sample, a magnification of images showing distortion of grid squares and a photograph of the THIM instrument.
