# Supplementary material for: Stationary beam full-field transmission helium ion microscopy using sub-50 keV He+: Projected images and intensity patterns
Source: Beilstein J Nanotechnol. 2019 Aug 7;10:1648–57. doi: 10.3762/bjnano.10.160 (PMC6693417; doi:10.3762/bjnano.10.160)

BEILSTEIN JOURNAL OF NANOTECHNOLOGY

Supporting Information

for

Stationary beam full-field transmission helium ion microscopy using sub-50 keV He^+: Projected images and intensity patterns

Michael Mousley, Santhana Eswara, Olivier De Castro, Olivier Bouton, Nico Klingner, Christoph T. Koch, Gregor Hlawacek and Tom Wirtz

Beilstein J. Nanotechnol. **2019,** *10,* 1648–1657. doi:10.3762/bjnano.10.160

Description of supporting videos 1 and 2, SIMION results and methods (defocus and size on sample plane), a defocus series for a polycrystalline silicon sample, a magnification of images showing distortion of grid squares and a photograph of the THIM instrument

License and Terms: This is a supporting information file under the terms of the Creative Commons Attribution License (http://creativecommons.org/licenses/by/4.0). Please note that the reuse, redistribution and reproduction in particular requires that the authors and source are credited.
The license is subject to the *Beilstein Journal of Nanotechnology* terms and conditions: (https://www.beilstein-journals.org/bjnano)

Supporting Information File 1 (BN_12point5kV_fire)
Video starts with focus on sample plane
Begin increasing lens 2 voltage
00:04 lens voltage overfocused to produce image of sample
Begin decreasing lens 2 voltage
00:05 underfocused spot pattern
Begin increasing lens 2 voltage
00:07 back to focus on sample
Decrease MCP voltage (lowers brightness)
Begin deceasing lens 2 voltage
00:11 Under focus spot pattern
00:16 second cross over as beam focus crosses MCP
00:19 beam focus behind MCP (extreme underfocus) low magnification projection image of grid
Begin increasing lens 2 voltage
00:22 underfocus spot pattern

Supporting Information File 2 (MgO_uncoated_10kV_fire)
Recorded for the uncoated MgO powder sample. Rapid small motion of spots is caused by instability of the beam which affected even a stationary beam with no sample

Starts at underfocus spot pattern
Lens2 voltage increased
00:04 beam focused on sample
00:07 overfocus deflection bright outline pattern
00:15 overfocus projection image of grid
Begin reducing lens 2 voltage
00:27 overfocus deflection bright outline pattern
00:39 underfocus spot pattern
Continue reducing Lens 2 voltage
00:52 Beam focused on MCP
00:56 extreme underfocus projection image , beam focus behind MCP
Begin increasing lens 2 voltage
00:59 beam focused on MCP
01:02 underfocus spot pattern

SIMION method and results

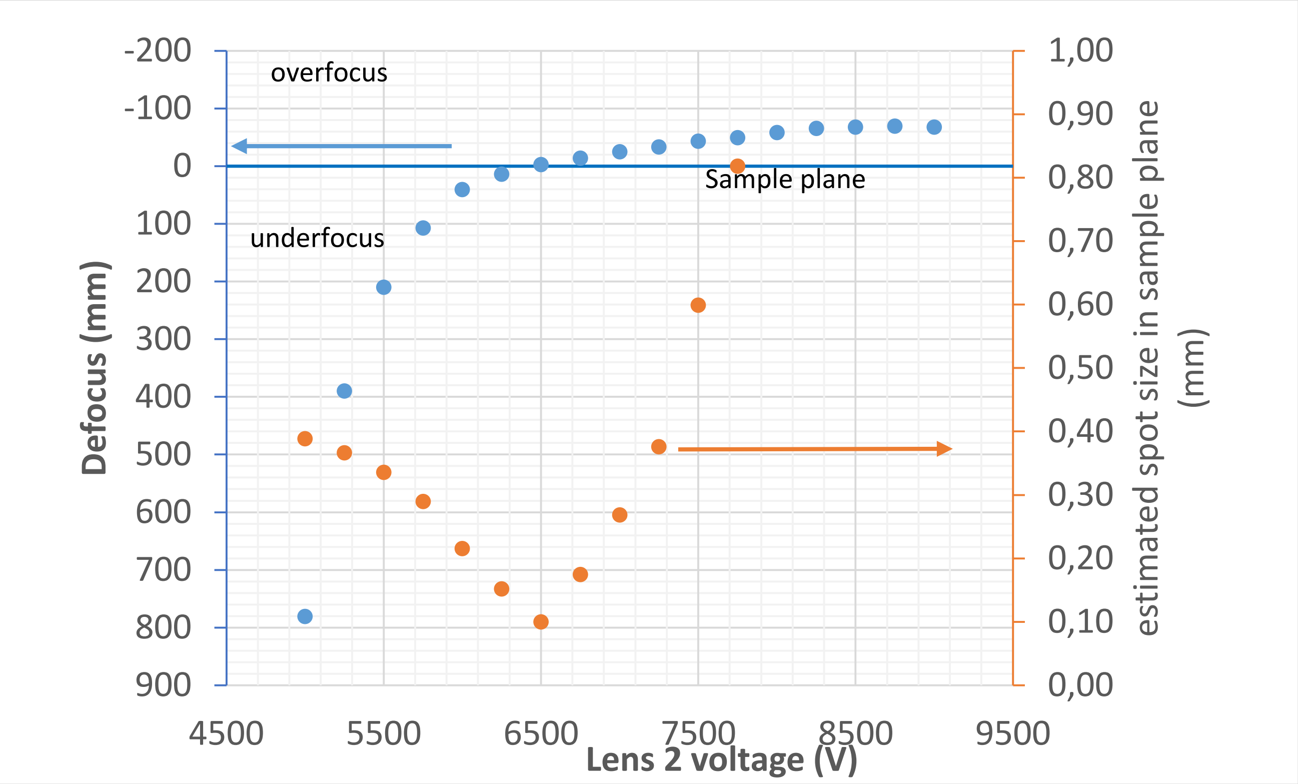

Figure S1: Simulated defocus values and estimated beam spot size in the sample plane.

The SIMION simulations calculated distributions of 10 keV He^+ ions. From these values the FW50 values were calculated, defined as the diameter of the circle enclosing 50% of the beam ions. The focal length, f ,was defined as the position of the minimum FW50. The defocus values (i.e., distance of the crossover with respect to the sample plane) have been calculated using the lens image equation $\frac{1}{d_o}+\frac{1}{d_i}=\frac{1}{f}$ where d_o is the distance from the lens to the object and d_i is the distance to the image. In this experiment the object is the source spot at the aperture, the image distance will be where the intensity density of the source image is highest. The d_o value was calculated by viewing a scanning secondary electron image, the best SE image resolution is when the lens 2 voltage focuses the source image on the sample plane. The known $d_i$and simulated f value for this best Lens 2 voltage were then used to find d_o, which was then used to calibrate the simulated focal lengths into d_i values.

The spot size estimates were done using the simulated defocus values (df), known distance to the sample plane and the measured diameter of sample intensity on the MCP (B) taken from some images of known Lens 2 voltage. These details were used, following Figure S2 to find values of C2 giving the same C1 for different df values. These were found to be

C1 = 70.6 mm and C2 = 157 µm for df = 0. These were then used to predict the size of the probe on the MCP, solve for the angle θ, and then calculate the spot size in the sample plane (orange double-headed arrow, S in Figure S2). These spot sizes are plotted on the right hand axis in Figure S1.

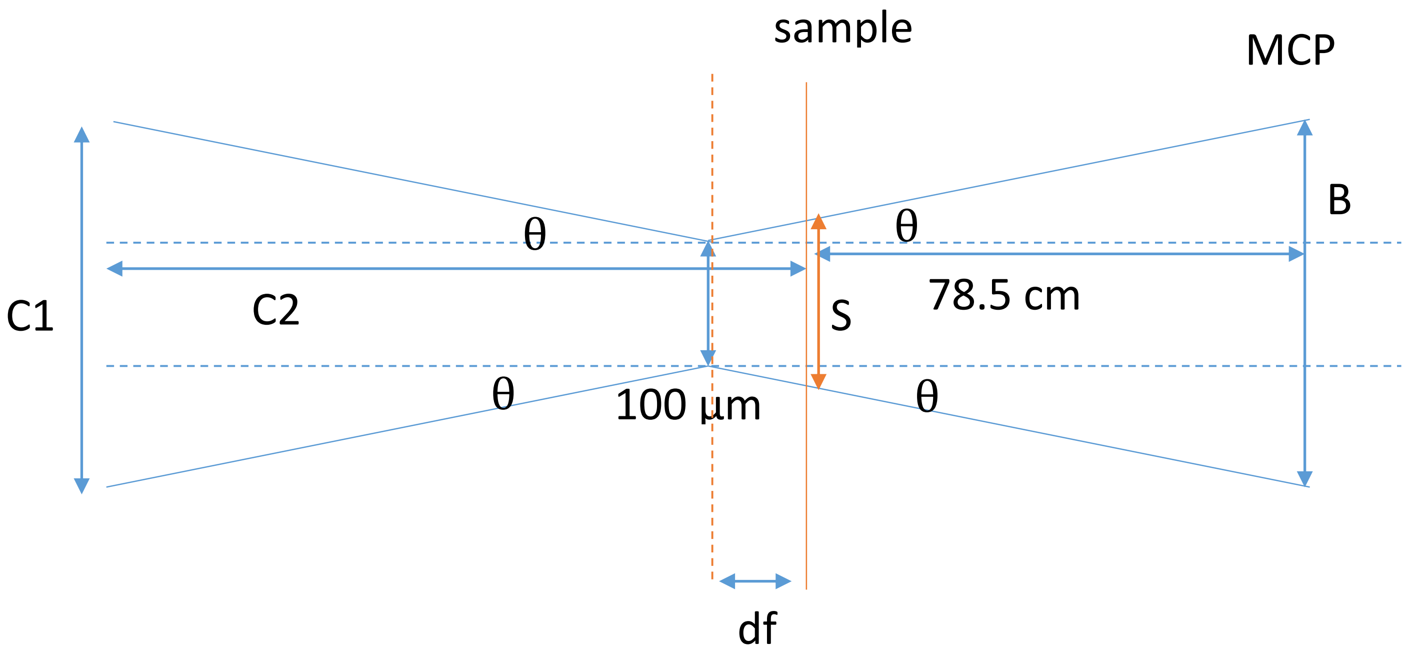

Figure S2: The schematic used for the estimation of the beam size in the sample plane.

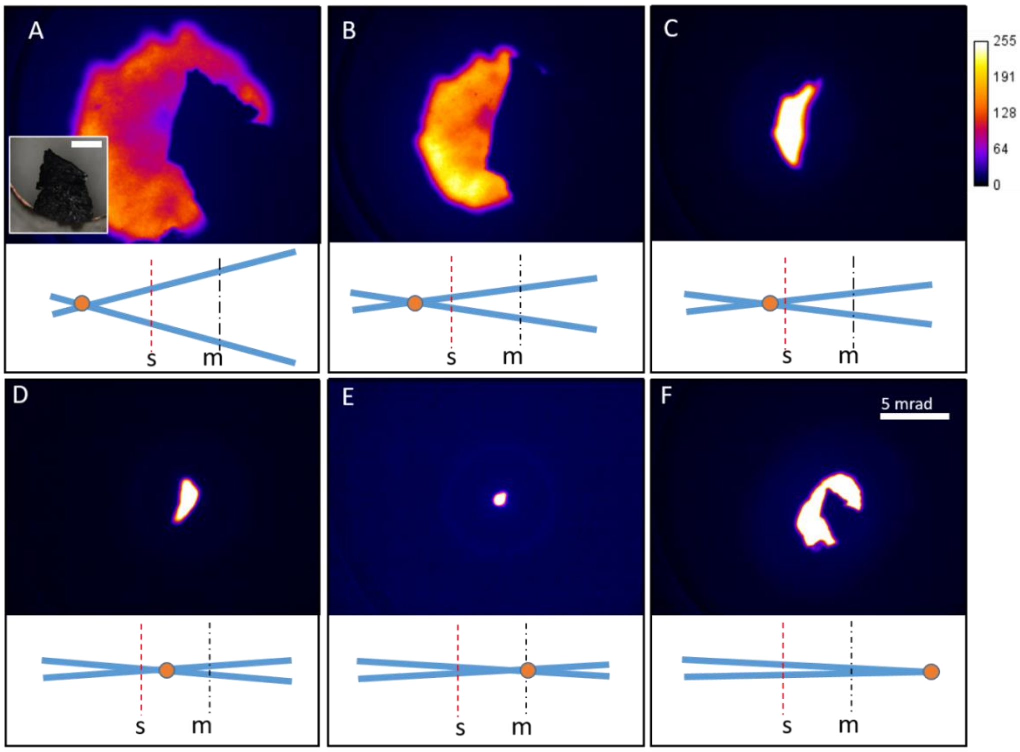

Figure S3: A) A THIM through focus series of a polycrystalline silicon sample produced by decreasing the lens 2 voltage from A to F. No deflection is visible, only inversion of the image occurs close to infinite magnification. The inset in A shows an optical image of the silicon sample, scale bar is 400 µm. Underneath each image is a lens diagram showing the position of the Lens 2 focal point cross over (orange circle), the dashed red line is the sample plane (labelled s) and the dot-dashed black line is the MCP plane (labelled m). The colourbar shows the grayscale pixel value recorded in the CCD image. The scale bar in F is 5 mrad and applies to all images. The bulk polycrystalline hyper pure Si samples (CAS #: 7440-21-3) were obtained from Wacker Chemie AG, Germany.

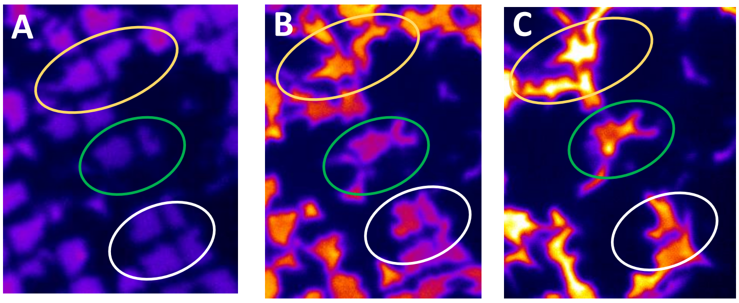

Figure S4: Examples of disortion of the grid square image as the focal point is brought closer to the sample plane (Lens 2 voltage increasing from A to C). All are overfocus images. Coloured ovals highlight intensity coming from idential sample regions for each image. The mesh was a copper 200 mesh, the grid hole size is 108 µm and the bar width is 19 µm (www.tedpella.com product 79750 Maxtaform Reference Finder Grids, Style H2, 200 mesh, copper).

Instrument Photograph

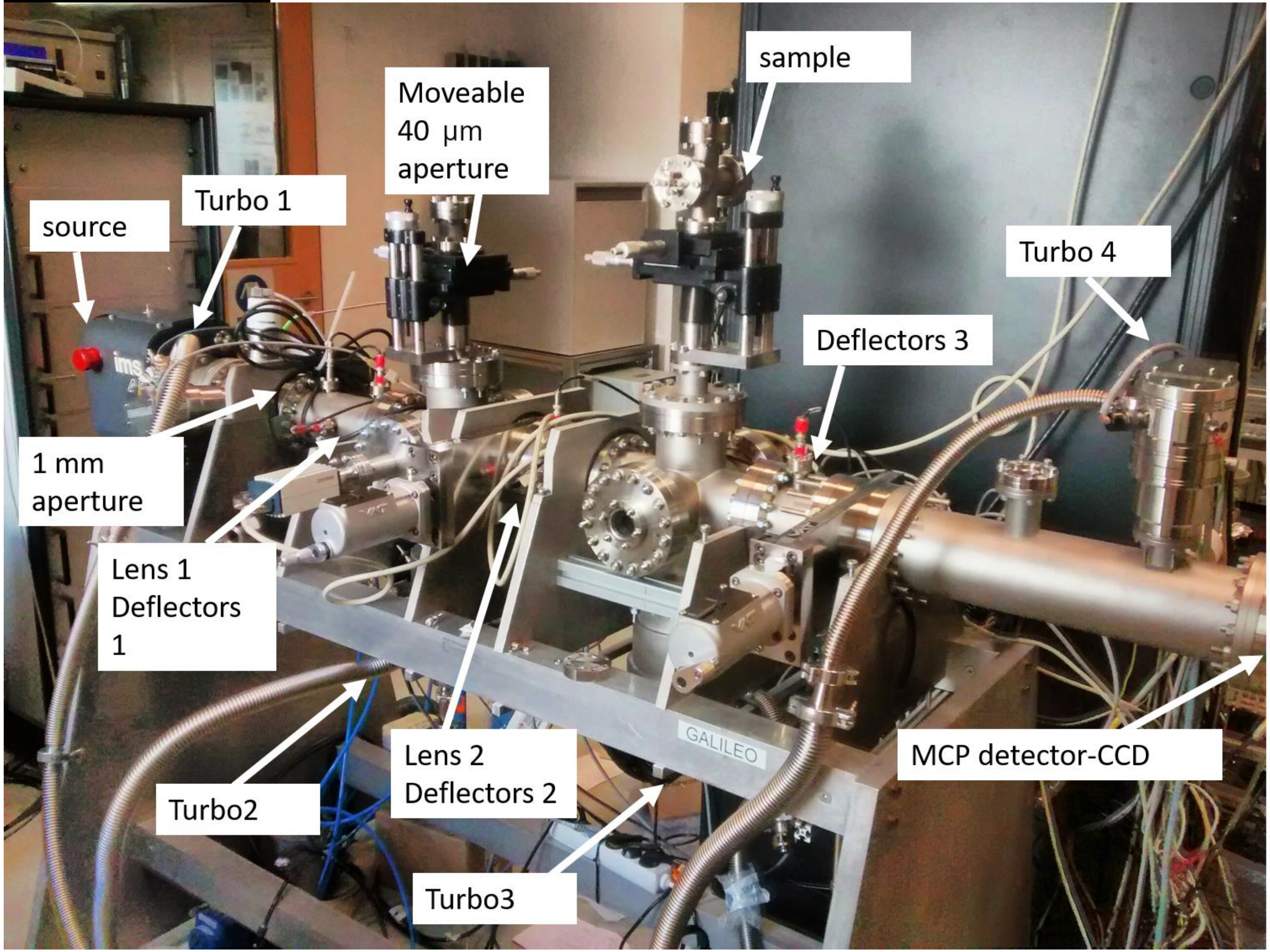

Supplement: File 3 — Description of supporting videos 1 and 2, SIMION results and methods (defocus and size on sample plane), a defocus series for a polycrystalline silicon sample, a magnification of images showing distortion of grid squares and a photograph of the THIM instrument. [file Beilstein_J_Nanotechnol-10-1648-s003.pdf]
